# Psoriasis, stem cells, and obesity: metabolic exploration for therapeutics

**DOI:** 10.25122/jml-2025-0033

**Published:** 2025-07

**Authors:** Carolina Constantin, Elena-Georgiana Dobre, Adriana Narcisa Munteanu, Mihaela Surcel, Constantin Căruntu, Sabina Andrada Zurac, Monica Neagu

**Affiliations:** 1 Immunology Laboratory, Victor Babes National Institute of Pathology, Bucharest, Romania; 2 Pathology Department, Colentina Clinical Hospital, Bucharest, Romania; 3 Department of Physiology, Carol Davila University of Medicine and Pharmacy, Bucharest, Romania; 4 Department of Dermatology, Prof. N.C. Paulescu National Institute of Diabetes, Nutrition and Metabolic Diseases, Bucharest, Romania; 5 Department of Pathology, Carol Davila University of Medicine and Pharmacy, Bucharest, Romania; 6 Doctoral School, University of Bucharest, Bucharest, Romania

**Keywords:** psoriasis, stem cells, immune cells, obesity, lipids, therapy

## Abstract

Psoriasis is a chronic inflammatory cutaneous disease with a complex pathogenesis that remains incompletely understood. New data suggest that psoriasis severity may be more accurately assessed by examining inflammation, oxidative stress, and hormones, although further research is needed to substantiate the clinical value of these biomarkers. The multifactorial causes of psoriasis encompass metabolic deregulations, such as lipid alterations that favor inflammation, exacerbate immune cell activity, and worsen the disease symptomatology. The pathophysiological link between psoriasis and obesity may be revealed through a crosstalk between adipocytes and the immune system, mediated by diverse soluble mediators, including adipokines. In this autoimmune disease, dermal mesenchymal stem cells (MSCs) are potential cellular players that connect autoimmune mechanisms, inflammation, and dysregulation of lipid metabolism. Networks of soluble factors, immune and non-immune cells, and MSCs mediate the inflammatory state in psoriasis. In many recent studies, the relapse has been associated with the potential role of MSCs in this process, endorsing MSCs as a new therapeutic reservoir in psoriasis. Thus, in our review, we aimed to evaluate the potential connection between autoimmunity, inflammation, and dermal mesenchymal stem cells, along with dysregulation of lipid metabolism, to elucidate the identity of psoriasis and identify potential new diagnostic and/or therapeutic targets.

## INTRODUCTION

Psoriasis (Pso) is a T-cell-mediated chronic inflammatory cutaneous disease characterized by dermal infiltration of immune cells and epidermal hyperplasia [1], with a complex pathogenesis that remains incompletely understood [2,3]. Pso incorporates an extensive range of cutaneous symptoms, including localized lesions or large plaques that can evolve into more extensive forms [4]. The presence of multiple distinct subtypes, the absence of reliable clinical biomarkers [4], and the intricate interplay of inflammatory and autoimmune mechanisms contribute to challenges in establishing accurate diagnoses and developing personalized therapeutic strategies [5].

New data suggest that the severity of Pso can be more accurately evaluated through markers of inflammation and oxidative stress, hormone levels, and even tumor-related markers. However, further extensive research is required to substantiate the clinical value of these biomarkers for disease management [4]. Prolonged inflammation and tissue damage found in Pso are sustained by an imbalance of innate and adaptive immunity that induces the expansion of the self-reactive lymphocytes and elevates the levels of autoantibodies, broadening autoinflammation [6,7]. The development of psoriatic plaques involves a complex interplay of immune mediators, including interleukins (IL)-17, IL-22, IL-23, and IL-10; immune cell subsets such as T helper 17 (Th17), T helper 22 (Th22), and regulatory T cells (Tregs); as well as key growth factors like transforming growth factor-beta 1 (TGF-β1) [8-10].

Keratinocytes and immune cells are increasingly recognized as key contributors to the multifactorial pathogenesis of psoriasis, engaging in bidirectional communication through specific cytokine–receptor pairs—a process that remains incompletely understood and continues to be actively investigated. Abnormal keratinocyte (KC) activity is a hallmark of psoriasis, following a well-characterized pathogenic sequence. Thus, KC becomes pathogenic under the influence of IL-17, tumour necrosis factor (TNF)-α, and IL-22, which are released by Th17 cells activated by IL-23 produced by dendritic cells. Among the immune cell populations that infiltrate Pso lesions, dendritic cells initiate and maintain the disease phases [11]. Additionally, elevated levels of pro-angiogenic factors and adhesion molecules contribute to the extensive recruitment and retention of immune cells within psoriatic plaques [12,13]. As recently reviewed by several groups, Pso encompasses not only an autoimmune basis but also metabolic components. Alterations in lipid metabolism exacerbate inflammation by modulating the function of immune cells, both systemically and locally, thereby amplifying disease severity. Dermal mesenchymal stem cells (MSCs) have emerged as key regulators at the intersection of autoimmunity, chronic inflammation, and lipid homeostasis. This review aims to explore the potential mechanistic link between these processes and to identify novel diagnostic or therapeutic targets in Pso.

## LIPID METABOLISM DEREGULATIONS IN PSORIASIS

The link between obesity and Pso has been largely documented in the last decade [14], with dyslipidemia identified as one of the critical metabolic alterations contributing to this cutaneous condition [15]. High levels of serum cholesterol, low-density lipoprotein cholesterol, triglycerides, or a reduced level of high-density lipoprotein cholesterol are trademarks for dyslipidemia [16]. Moreover, the adipose tissue, through its increased production of adipokines and cytokines (mainly IL-6 and TNF-α), exerts significant effects on immune cells' responses and drives the course of inflammation. The interconnection between Pso, obesity, and dyslipidemia is a subject of intense research for the future development of both diagnostic markers and therapeutic targets [17,18]. This triad can be examined from multiple perspectives. Obesity exacerbates many inflammatory cutaneous disorders, including Pso, by altering the immune condition *through* hypertrophic adipocytes, which lead to a deregulated secretion of both pro- and anti-inflammatory cytokines [19]. Key factors in obesity-induced inflammation include several adipokines (e.g., leptin, resistin, adiponectin), with their serum levels and corresponding receptor expression in adipose tissue serving as indicators of the obesity–psoriasis connection. The role of leptin in the obesity–Pso connection has been underscored by several studies, which have consistently reported elevated leptin expression and circulating levels in patients with Pso compared to controls [20]. Moreover, in obese individuals, increased levels of serum leptin trigger the release of TNF-α and pro-inflammatory factors (IL-1, IL-6, IL-17, IL-22) contributing to the onset of Pso by inducing KC proliferation, skin layers hyperplasia, and angiogenesis [21,22]. Another adipose-tissue-related cytokine with a core role in the obesity-Pso connection is adiponectin. Its decreased serum levels in obese patients with Pso are linked to an increased severity of skin lesions [23].

Within adipose tissue, a complex system of stromal vascular cells, populations that mediate chronic inflammation, actively contributes to metabolic deregulation [24]. Of particular interest are macrophages derived from adipose tissue, which, beyond their physiological roles (e.g., tissue remodeling), are actively recruited during the progression of obesity. These cells migrate from the bloodstream into fat tissue, where they proliferate and enter a complex activation state that alters adipocyte function and induces inflammation in adipose tissue [24]. As a result, certain inflammatory cytokines such as IL-17 and TNF-α are produced, favoring the expansion of Th17 cells that drive Pso pathogenesis [25,26].

Similarly, Langerhans cells (LCs) exhibit metabolic dysfunctions that trigger various skin pathologies [27] and display a specific pattern of pro-inflammatory cytokines. A recent study has shown that LCs isolated from psoriatic lesions display altered lipid metabolism, which impacts both their immune response and immune surveillance functions as antigen-presenting cells (APCs) in the skin. There is an increased lipid content of psoriatic LCs due to the impairment of lipid autophagy. This process is linked to a high IL-23p19 messenger ribonucleic acid (mRNA) level, which stimulates IL-23 secretion. This specific metabolic phenotype of LCs contributes to the pathogenesis of Pso and also highlights potential metabolic targets for novel therapeutic strategies [15].

Nutrition is an environmental factor and an external trigger that alters lipid metabolism in Pso. It was reported that compared to normal individuals, patients with Pso have more often altered dietary habits like a higher consumption of fat, leading to an increased severity of Pso as well as development of various comorbidities. Dietary lipids are essential nutrients, with fatty acids being the primary components. These fatty acids vary in chain length and degree of saturation, broadly categorized into short-chain fatty acids (SCFAs) and unsaturated fatty acids, such as omega-3 and omega-6 polyunsaturated fatty acids (PUFAs). A high-fat diet enriched in free fatty acids (FFAs) might aggravate Pso. At cellular level, FFAs sensitize dendritic cells (DCs) to amplify Th1/Th17-immune responses in obesity-related pathologies such as Pso [28]. Thus, FFAs stimulate skin myeloid DCs to produce proinflammatory cytokines (e.g., IL-1β), which further activate KCs. The activation of KCs is mediated by the release of chemokines that induce the recruitment of neutrophils and monocytes, thereby intensifying the inflammatory response at the lesion site. In addition, another effect of FFA on DCs is to activate these cells, which further induce Th1/Th17 differentiation, thereby aggravating the psoriatic condition [29]. Thus, many studies conclude that a high-fat diet intensifies early psoriatic skin inflammation, and saturated fatty acids are amplifiers of psoriatic dermatitis [17].

Lipid imbalances can disrupt finely regulated intracellular systems, including the inflammasome—a large multiprotein complex that plays a key role in the progression of autoimmune diseases. Numerous studies have identified the inflammasome as a danger-sensing mechanism in KC, contributing to the cutaneous inflammatory responses characteristic of Pso [30,31]. SFAs have been reported to exacerbate Pso and its related comorbidities by activating the nucleotide-binding oligomerization domain (NOD)-like receptor family pyrin domain-containing 3 (NLRP3) inflammasome and promoting the TNF-α/IL-23/IL-17 inflammatory axis, as well as by suppressing the function of regulatory T cells (Tregs). Conversely, *n*-3 PUFAs can ameliorate the psoriatic condition by suppressing inflammatory pathways or by inducing Treg activity [32,33]. The link between SFAs and Pso was emphasized in a study with murine models of psoriatic dermatitis. Thus, it was discovered that SFAs aggravate the disease by activating the inflammasome in both KCs and macrophages, inducing expansion of Th17 and IL-17-γδ T cells in the skin. In contrast, omega-3 PUFA have been shown to suppress Th17 cell differentiation. These intricate interplays between skin cell populations and lipids reveal new perspectives for therapeutic strategies and/or alleviating disease symptomatology [34].

The modulation of lipids, as important blocks of immunometabolism, emerges as a promising adjuvant therapeutic target in Pso. The type of response, the involved cellular subtype, and the secreted cytokine set indicate that T cells are crucial players in Pso development. However, the metabolic inquiries in different T-cell subsets are less investigated and therefore might pave the way for a new understanding of Pso pathogenesis. An integrated analysis of transcriptomic features, inflammation, and metabolic landscape of peripheral T lymphocytes subsets in Pso patients has recently revealed that fatty acid catabolism may suppress Tregs activity. Thus, the inflammatory responses of CD4^+^ central memory T cells and CD8+ effective memory T cells are blocked by an active metabolism of alpha-linolenic acid, linoleic acid, and arachidonic acid in this type of disease [35].

A significant consequence of lipids deregulation in Pso is the appearance of lipids antigens in the specific lesions produced by mast cells that carry a high content of cytosolic phospholipase A_2_ (PLA2) [36]. Lipid antigens formed by mast cells are presented by LC to T cells, sustaining the activation loop. In this sense, recent evidence has identified LC with constitutive high expression of CD1a that endorses lipid antigen presentation. In addition, it was suggested that not only PLA2 is involved in CD1a neolipid antigen generation in Pso, but a broader spectrum of enzymes could participate in this process. For instance, lipase acyloxyacyl hydrolase (AOAH) acts similarly to PLA2 [37], potentially activating circulating CD1a auto-reactive T cells that produce elevated levels of interferon (IFN)-γ and IL-22. Therefore, therapeutic inhibition of AOAH activity would be beneficial in Pso, as it controls the aberrant levels of IFN-γ and IL-22 in psoriatic inflammation [38,39].

As AOAH is found in low levels in circulating neutrophils and neutrophil’s abundance in the lesions is a distinctive histopathological sign of the Pso condition [40], it was suggested that the high number of neutrophils sustains an increased expression of AOAH in psoriatic lesions; thus, the level of AOAH could serve as a marker for evaluating CD1a-reactive T cell responses in Pso [38].

Neo-lipid antigens controlled by CD1a^+^ LCs *via* exosomes are further recognized by so-called self-lipid-specific CD1a-reactive T lymphocytes. This interaction promotes the release of IL-22 and IL-17A in large amounts, a process that is highly unfavourable for Pso lesions. Studies in mice models have reiterated that fluctuations in lipid levels link CD1b-restricted T lymphocytes to autoimmunity and contribute to hyperlipidemia-induced cutaneous inflammation [41]. Therefore, in Pso, the network of adipocytes, lipid metabolism molecules, and immune cells could trigger and further enhance psoriatic lesions.

## AUTOANTIBODIES IN PSORIASIS

Autoantibodies in Pso have been extensively reviewed in recent studies [42,43], highlighting their potential role in disease severity and progression. Anti-citrullinated protein antibodies (ACPAs) and antibodies targeting mutated citrullinated vimentin (MCV) have been associated with more severe manifestations of both Pso and psoriatic arthritis (PsA) [44]. Antinuclear antibodies (ANAs) are significantly associated with the type of Pso [45] and can help detect the efficacy of therapy in Pso [46]. Autoantibodies generated against inflammatory regulators, such as calpastatin (immunoglobulin G anti-calpastatin antibody), were found to be elevated in 27% of Pso patients [47]. Interferon-inducible protein 16 (IFI16), an innate immune sensor, has been implicated in the pathogenesis of Pso and PsA, with autoantibodies against IFI16 reported at elevated levels in patients with both conditions [48]. Similarly, autoantibodies targeting heat shock protein 90 (Hsp90) have been identified in Pso patients, with levels positively correlating with disease severity [49]. In the context of lipid metabolism, increased titers of autoantibodies against oxidized low-density lipoprotein (ox-LDL) have been observed, showing a significant association with higher Psoriasis Area and Severity Index (PASI) scores [50]. Beyond these systemic autoantibodies, those directed against skin-specific antigens are also characteristic of Pso. Antibodies against keratinocytes integrin, namely against alpha6-integrin, were found in 30% of Pso patients [51]. Antibodies against LL37 (a cationic antimicrobial peptide overexpressed in Pso epidermis) and against ADAMTSL5 (A Disintegrin-Like and Metalloprotease Domain Containing Thrombospondin Type 1 Motif-like 5) are antibodies that sustain the inflammatory pattern of Pso and are associated with disease severity. [52]

### Autoantibodies and lipid metabolism

There are two sides to the relationship between autoantibodies and lipid metabolism in autoimmune diseases. One approach targets the increased lipids, and the other involves the existence of lipid-based autoantigens. In Pso, like in other autoimmune diseases, the treatment is focused on a specific target, maintaining immunosuppression. However, nutrition plays a crucial role in the prevention and management of autoimmune diseases [53]. Dietary interventions can influence disease progression, severity, relapse, and even the occurrence of adverse events induced by pharmacotherapy [54]. As previously stated, overweight and obesity are common conditions; hence, a comprehensive dietary approach is necessary [30]. Interestingly, in the management of autoimmune diseases, the low target specificity can induce unwanted side effects on cellular metabolism [55]. Upon medications, many patients can develop hyperlipidemias and cardiovascular (CV) risk [56]. To alleviate this side effect, alternative metabolic pathways are being investigated, and anti-ceramide therapies may aid in autoimmune diseases [57]. The other facet of auto-antibodies linked to lipid metabolism is the existence in Pso patients of various autoantibodies, including lipid antigen phospholipase A2 group IVD (PLA2G4D) [58]. Mast cells within psoriatic lesions actively secrete PLA2G4D, generating neo-lipid antigens that are processed by LCs. This interaction leads to the production of pro-inflammatory cytokines IL-22 and IL-17A [59]. Autoreactive T cells specific for lipid antigens sustain the psoriatic inflammation in Pso patients that lack other auto-reactive T cells against LL-37 or ADAMTSL5 [60].

The presence of various autoantigens, such as LL-37, ADAMTSL5, and keratin 17, accompanies the lipid-derived PLA2G4D in psoriatic skin. These antigens can generate specific autoreactive T cells, and thereafter B cells can generate autoantibodies. Studies evaluating autoantibodies in patients with Pso have demonstrated significantly elevated levels of anti-gliadin IgG4, whereas autoantibodies against heterogeneous nuclear ribonucleoprotein (hnRNP)-A1 and ADAMTSL5 did not differ significantly from healthy controls. Given that gliadin is a dietary antigen, these findings raise the question of whether specific nutritional components might act as triggers for autoimmune responses in Pso [61,62].

## NEW CELLULAR LINKERS BETWEEN PSORIASIS AND OBESITY – MSCs

Several clues suggest that MSCs are involved in maintaining the delicate balance between autoimmunity, inflammation, and lipid metabolism. Hence, MSCs in a psoriatic condition have a high secretion of pro-inflammatory molecules and display markers that indicate their involvement in autoimmunity. Furthermore, adipose-derived MSCs, including those from subcutaneous fat, have been shown to trigger inflammatory signaling pathways in psoriatic conditions. Thus, in search of the cellular culprit that links autoimmunity to inflammation and lipid metabolism, MSCs can be one of the perpetrators.

### Mesenchymal stem cells and autoimmunity

MSCs are multipotent adult cells with self-renewal, increased proliferation, and the capacity to differentiate into cells associated with mesenchymal tissues from various anatomical sites, including bone, fat, and cartilage [63]. These properties render MSCs as a promising cell source for biomedical applications, particularly in the field of tissue regeneration [64]. The most enriched source of MSCs that is appealing to biomedical investigations is the adipose tissue, bone marrow, placental tissues, and umbilical cord, where MSCs possess different pluripotent and regulatory capacities [65]. *In vitro*, MSCs are morphologically similar to adherent fibroblasts and have the potential to form colonies [66]. According to the International Society for Cellular Therapy (ISCT), the minimal criteria for defining MSCs include adherence to plastic surfaces under standard culture conditions, expression of surface markers CD105, CD90, and CD73, and the absence of hematopoietic markers such as CD14, CD34, and CD45 [67].

In addition to phenotypic characterization, the nomenclature of MSCs has been a topic of considerable debate—particularly regarding the use of the term 'stem' versus 'stromal'. To address this issue, ISCT recommended the adoption of the term 'mesenchymal stromal cells'. Furthermore, ISCT advocates for the incorporation of secretome profiling and functional assays to supplement phenotypic markers, thereby homogenizing regulations regarding their biomedical applications [68,69]. These clarifications aim to explain the difficulties in corroborating different results from various research groups, where the same cells were reported under different designations.

Under specific conditions, MSCs can differentiate into diverse mesenchymal cell lines (e.g., osteoblasts, chondrocytes, adipocytes, endothelial cells, and cardiomyocytes) as well as non-mesenchymal cell lines (e.g., hepatocytes and neuronal cell types) [70].

A 2019 study assessed the functional capacity of dermal and epidermal MSCs in patients with psoriasis, focusing on morphology, immunophenotype, and differentiation potential. The authors confirmed the previously described phenotype, and MSCs from both dermal non-lesion and psoriatic lesions exhibited higher human leukocyte antigen (HLA)-I expression compared to healthy dermis. Additionally, a decreased T cell immunosuppression capacity was observed in the non-lesional dermis, demonstrating that MSCs may favor a skin psoriatic condition [71].

The multipotency of MSCs implies an extensive coverage of biological functions such as inter-cellular communications, regulation of the surrounding microenvironment, and homing to injured tissues to accelerate repair/regeneration. These functions are mediated by a repertoire of bioactive molecules secreted by MSCs, such as chemokines, cytokines, and growth factors—including granulocyte-macrophage colony-stimulating factor (GM-CSF), leukemia inhibitory factor (LIF), stem cell factor (SCF), thrombopoietin, and interleukins (IL-8, IL-10, IL-11, IL-14, IL-15) [72,73]. The MSC secretome comprises not only soluble factors (e.g., cytokines and growth factors) but also extracellular vesicles enriched with microRNAs (miRNAs), messenger RNAs (mRNAs), and proteins, enabling robust paracrine activity [74,75]. These paracrine effects are central to the therapeutic utility of MSCs in regenerative medicine and may also involve their role in regulating autoimmune responses [76-79]. As autoimmunity is an abnormal, complex, and chronic condition in which immune cells lack tolerance to self-antigens and attack the body's cells/structures, therapeutic efforts are challenging. One should consider both symptom severity and the cause of immune deregulation in psoriatic conditions. In recent years, MSCs have been considered as alternate tools in treating and decoding autoimmune disorders due to their unique immunomodulatory functions and secretory capacity tailored to the inflamed milieu [80]. The difficulties related to standard anti-inflammatory drugs can be surmounted by strategies able to induce and maintain a tolerogenic status. In this scenario, MSCs could be exploited *through* their cross-talk processes with DCs and Tregs, key cells responsible for restoring immune tolerance [81].

Recently, the effect of psoriatic dermal mesenchymal stem cells (p-DMSCs) on the functions of T cells, including proliferation, apoptosis, and differentiation, has been investigated. p-DMSCs and DMSCs from healthy volunteers, isolated from psoriatic and normal skin and co-cultivated with activated normal T cells, were assessed to evaluate proliferation and apoptosis of T cells. One study investigated the expression of cytokines and transcription factors associated with various T cell subtypes using qRT-PCR and Western blotting. The authors demonstrated that both psoriatic and normal DMSC inhibited T cell proliferation and cytokine production. Furthermore, a reduction in the expression levels of the transcription factors T-bet and RORγt was observed in T cells, with normal DMSCs exerting a stronger inhibitory effect than psoriatic DMSCs. These findings suggest that DMSCs may contribute to the pathogenesis of Pso by modulating T cell responses [82].

### Stem cells and obesity

There are clear differences in the cellular profile of human stem cells derived from obese individuals compared to those from lean individuals. Over the last decade, reports have highlighted several connections between obesity and the function of MSCs [83]. In a study comparing the immunophenotypic profile and plasticity of human adipose-derived stem cells (hASCs), differences were revealed depending on their source, specifically between lean- and obese-derived cells. Thus, obese-derived hASCs exhibit increased proliferation and migration capacity, lower CD29 expression, and higher HLA-II and CD106 expression. Additionally, hypoxia increases the proliferation and migration of lean hASCs, altering the expression of CD36 and CD49b. This suggests that obesity significantly conditions stem cell behavior, and that the altered plasticity observed in hASCs from obese individuals may be independent of oxygen tension, implying a lasting imprint of the obese microenvironment on stem cell function and differentiation potential [84].

The pathophysiological link between Pso and obesity is mediated by a complex cross-talk between adipocytes and the immune system, primarily through various adipokines. In obesity, adipocytes exhibit upregulated expression of pro-inflammatory adipokines, such as leptin and resistin. Furthermore, macrophages in obesity-associated conditions tend to release additional pro-inflammatory cytokines, thereby amplifying an already established state of systemic hyperinflammation [85].

Adipose tissue is now increasingly scrutinized for its metabolic and endocrine functions, rather than just being viewed as a fat storage depot. In this scenery, adipose-derived stem cells, a subset of MSCs from adipose tissue, have been demonstrated to possess regenerative and immunological functions, having a key role in regulating both adipocyte function and skin regeneration [74,86].

As another recent facet of Pso, numerous studies focusing on lipidomic approaches have linked MSCs to a worse prognosis for Pso. Several distinct cellular populations are implicated in Pso relapse, including skin-resident memory T cells (TRMs), memory-like γδ T cells, KCs, and LCs. Thus, the recurrence of Pso was described by a mechanistic model, in which the distinct localization and behavior of immune and non-immune cells in the skin layers explain the differences between clinically resolved lesions and damaged skin. Hence, in clinically cured lesions, CD4^+^ TRMs are in the dermis, whereas CD8+ TRMs and LCs append to the epidermis. LCs produce IL-23 and stimulate the release of IL-22 from CD4^+^ TRMs and trigger KC hyperproliferation and inflammation. Additionally, CD49-CD8+ TRM cells produce IL-17 that sustains recurrent local inflammation. Simultaneously, effector memory T cells interact with vascular E-selectin, migrate to the skin, and release IL-17, contributing to disease relapse [87].

An increasing number of studies indicate that lipid metabolism plays a crucial role in maintaining the integrity of the skin barrier. Several reports have indicated that deregulations of lipids in Pso could be revealed at different levels, such as the presence of abnormal bioactive lipids in psoriatic lesions. Of these, sphingolipids may exert a significant effect on KC growth in Pso [87-89], while some interferences with Th17 cells are caused by lipid metabolite deregulation, designating Pso as an immuno-metabolic condition [86-90].

### Stem cells in psoriasis relapse

The multifactorial nature of Pso is a common feature of both its onset and relapse. Pso recurrence is influenced by both genetic predisposition and non-genetic factors, including skin and gut microbiota dysbiosis, dyslipidemia, and infections [17]. The Pso relapse is an intensely studied subject, as MSCs can play a key role in this process. Thus, certain genes and their corresponding proteins, linked to inflammation and angiogenesis, were assessed using omic technologies in dermal MSCs. The omics analysis revealed that in dermal MSCs, lipopolysaccharide-induced tumor necrosis factor (LITAF), hematopoietically expressed homeobox protein (HHEX), and dual-specific phosphatase (DUSP)1 expression levels were significantly lower in Pso patients compared to controls. This data suggests a role for dermal MSCs in the onset, development, and modulation of the inflammatory milieu in psoriatic lesions [91]. However, despite a clinical remission in up to 60% of patients receiving biological therapy, Pso may recur after withdrawal of systemic immunomodulatory treatments. In this frame, the relapse-stem cell cluster was analyzed, along with a form of 'inflammatory memory' depicted in resolved Pso skin, which may underlie the mechanism of Pso recurrence following systemic drug cessation. Novel data links immune and non-immune cells to the disease recurrence and inflammatory memory. In addition, the balance between Pso remission and recurrence has become a substrate for personalized therapeutic tactics, with ongoing research addressing this complex interplay [92]. Conversely, Pso relapse appears to be underpinned by a reactivation of key immune cells within the disease [93]. Thus, immune cells, such as the skin’s TRMs, memory-like γδT cells, and non-immune cells, like KCs and fibroblasts, appear to reinitiate the lesion based on their immune-inflammatory memory. However, the precise mechanisms underlying this process remain unclear [94,95]. Recent studies have suggested that a KC-cellular prototype from the skin basal layer, known as skin epithelial stem cells (EpSCs), plays a role in disease relapse; these EpSCs may acquire memory during inflammation, affecting psoriatic skin. These results indicate stemness-epidermal cells as contributors to lesion recurrence [94]. Moreover, in a Pso-like murine model induced by imiquimod (IMQ), Fuchs *et al*. suggested that EpSCs are direct responders to inflammation, have an expanded life and an improved wound healing capacity in psoriatic mice compared to controls [96,97].

### MSCs as new therapy sources for Pso

Ongoing research is exploring innovative strategies to alleviate Pso, with MSC-based therapies emerging as a promising avenue. One proposed mechanism suggests that MSCs, in coordination with matrix metalloproteinase-13 (MMP-13), may inhibit the aberrant activation of KCs characteristic of Pso. MMP13 plays a critical role in extracellular matrix remodelling, and its expression is found to be elevated in psoriatic skin lesions of an IMQ-induced murine model [98]. In this model, the study concluded that intravenously infused human umbilical cord MSCs (hUC-MSCs) can lead to MMP13 downregulation that inhibits KC proliferation, an important therapeutic outcome in psoriatic condition. They further demonstrated that MMP-13 upregulation is driven by TNF-α, which modulates the nuclear factor kappa B (NF-κB) signaling pathway in KCs. The decrease in MMP13 expression under the influence of MSCs opens new possibilities for therapeutic modulation in Pso [98]. Similar results were obtained in another mouse model of Pso-like inflammation where MSCs pre-activated by TNF-α/IFN-γ displayed therapeutic efficacy by decreasing neutrophil recruitment in psoriatic lesions; this effect seems to be mediated by tumor necrosis factor-alpha-stimulated gene/protein (TSG)-6 gene by reducing the expression of CXCL1 (C-X-C Motif Chemokine Ligand 1) responsible for neutrophil homeostasis and recruitment at the lesion site [99]. Hence, lesions could be alleviated via MSCs that impede neutrophil accumulation in Pso, revealing another possible cellular-based therapeutic approach [100]. To explore the MSCs dimension in Pso, an equivalent study in a mouse model of IMQ-induced psoriatic-like lesions revealed that subcutaneous injection of hUC-MSCs decreases the severity of Pso lesions. The clinical results matched the flow cytometry pattern of certain Th, Treg, and γδ T analyzed cells; thus, a reduction in skin inflammation was correlated with a decrease in the level of IL-17-producing γδ T cells [101,102]. These findings reinforce the utility of animal models in advancing our understanding of MSC-based therapies and support their translational relevance in the clinical management of Pso.

Early clinical trials have also been initiated. A phase 1/2a, single-arm clinical study evaluated the safety and efficacy of intravenous hUC-MSC infusions in 17 patients with Pso. No adverse events were reported during treatment or the subsequent six-month follow-up period. The follow-up revealed that the efficiency was higher in women (66.7%) compared to men (25%), and the overall effect showed that over 47% of the enrolled patients registered an improvement in the PASI score. Additionally, all responders exhibited substantial increases in Tregs and CD4^+^ memory T cells. Th17 cells and serum IL-17 levels were significantly decreased after therapy, suggesting that Treg levels may be a helpful indicator for predicting the clinical efficacy of the hUC-MSCs procedure [103].

**Figure 1:**
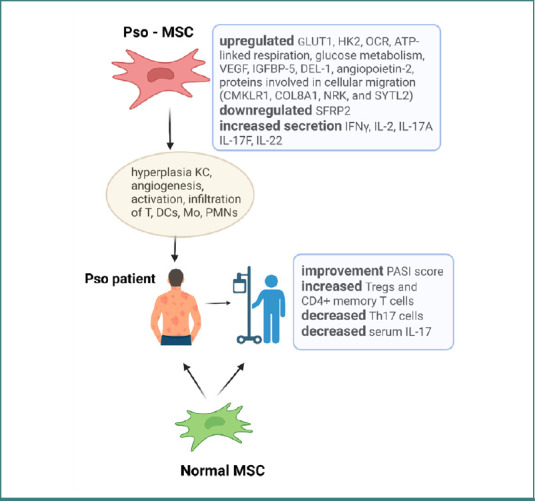
MSCs' involvement in Pso pathology and therapy. Pso-MSCs can sustain the pathology by inducing hyperplasia of keratinocytes, angiogenesis, and the activation of immune cells, including T lymphocytes, dendritic cells, monocytes, neutrophils, basophils, and eosinophils. Normal MSCs, when infused in Pso patients, can restore symptomatology by lowering the psoriasis area and severity index (PASI score), increasing Tregs and CD4+ memory T cells, and decreasing Th17 and serum IL-17 levels.

An outline of MSCs sources, involvement in skin autoimmune and inflammatory pathology, and their possible therapeutic impact is depicted in Table 1 and Figure 1 [104-115].

**Table 1:** MSCSs origins and their therapeutic applications

MSC source	Therapeutic effects	References
Mouse bone marrow	Suppresses T cells, B cells, IgE	[104]
Rat bone marrow	Ameliorates psoriasis-like inflammatory changes in the skin	[105]
Human umbilical cord	Diminishes the severity of IMQ-induced psoriasis-like dermatitis	[106]
	Reduces the production of IFN-I by pDCs	[107]
	Alleviates psoriasis through the TNF-α/NF-κB/MMP13 pathway	[98]
	Reduces psoriasis symptoms including thickness, erythema, and scales of skin lesions; increases Th2 cells; and reduces enrichment of inflammatory cytokines (IL-17A, IFN-γ, IL-6, TNF-α) in spleen and skin lesions	[12]
Human umbilical cord blood	EGF secreted by hUCB-MSCs can improve atopic dermatitis (AD) by regulating inflammatory responses of keratinocytes, Th2 cells, and mast cells	[108]
	Ameliorates AD by inhibiting secretion of TNF-α and IgE	[109]
Mouse AD skin	Ameliorates AD by suppressing the IL-17 expression of Th17 cells	[110]
Human AD skin	Improves Pso-like skin inflammation in mice by negatively regulating ROS	[111]
Human tonsil	Improves inflammatory skin lesions in mice; inhibits T-cell and B-cell mediated responses, and enhances the anti-inflammatory responses	[112]
Human gingiva	Attenuates IMQ-induced murine Pso-like skin inflammation	[113]
Human exfoliated deciduous teeth	Attenuates AD-like skin lesions in mice by modulating the immune balance and skin barrier function	[114]
Human dental pulp tissues	Reduces the symptoms of skin lesions and suppresses local and systemic immune responses of IMQ-induced Pso in mice	[115]

Tregs play a crucial role in maintaining immune homeostasis and preventing autoimmune conditions by suppressing excessive immune reactions. Their suppressive function is impaired in Pso, leading to an imbalanced T-h17/Treg ratio. The aggravation of Pso lesions is linked to Treg dysfunction; however, the precise regulation of Treg function is still poorly understood [116]. Tregs could be a candidate for systemic cellular therapy, but one important limitation for their use in the clinic is related to their phenotype transition, which alters their functions and efficacy. Thus, in a Pso-IMQ-induced mouse model, Zhang *et al*. [117] have recently approached the capacity of Treg in treating Pso from a metabolic perspective. Hence, increasing fatty acid level in the Pso inflammatory milieu would raise *Foxp3* expression in Tregs, restoring their suppressive functions and alleviating Pso symptoms [8]. Similar research performed by Schwarz *et al*. [118] hypothesized that gut microbiome is an important immunomodulator of Tregs through SCFA derived from bacterial fermentation [101]. It was presumed that sodium butyrate (SB), one of the SCFA products, could modulate Tregs activity in the skin. SB topically applied to psoriatic skin in a mouse model restored the number of Tregs and their suppressive function. Restored Treg levels were found in both blood and psoriatic lesions, a process that resulted in IL-17 downregulation and upregulation of IL-10 and forkhead box P3 (FOXP3) protein transcripts, associated with a reduction in inflammation [118].

The secretome machinery of MSCs, comprising exosomes (nanovesicles), microvesicles (ectosomes), and large extracellular vesicles (e.g., apoptotic bodies), could be explored in terms of MSCs as potential new therapy providers. This 'soluble' component could serve as an adequate acellular therapeutic substitute, offering reduced immunogenicity and potential as a druggable delivery system to specifically target dysfunctional cellular pathways [119].

## A FUNCTIONAL TRIAD: PSO-MSCS AND IMMUNE CELLS

**Figure 2:**
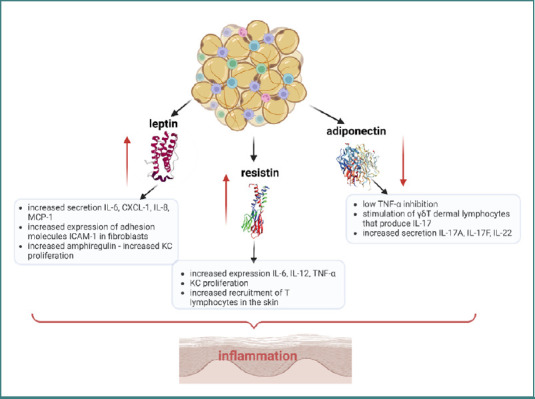
The interrelation of the main adipokines with pro-inflammatory cytokines and their effect upon inflammation in Pso

Angiogenesis represents a physiological process linked to wound healing, inflammation, as well as tumor development and the pathogenesis of Pso. In Pso-MSCs, angiogenic activity has been observed, with new vessel formation serving as a pro-inflammatory mechanism that facilitates leukocyte recruitment from the periphery into psoriatic skin. High expression of the pro-angiogenic mediator vascular endothelial growth factor (VEGF) has been reported in MSCs derived from psoriatic lesions compared to those from healthy skin, which sustains angiogenesis and maintains inflammation [120]. Supporting this mechanism, a proteomic analysis of molecules involved in cellular migration in healthy peripheral blood mononuclear cells (PBMCs) identified a panel of proteins (CMKLR1, COL8A1, NRK, and SYTL2) exhibiting upregulated expression in Pso-MSCs [121]. This MSCs phenotype was confirmed by similar studies where adipose tissue-derived MSCs exerted pro-inflammatory actions when driven by a pathological condition. Thus, signals released by MSCs to attract inflammatory immune cells nearby further generated an inflammatory landscape maintaining MSCs’ dysregulated behavior [120]. In a study performed several years ago, it was observed that Pso-MSCs were ineffective in suppressing T lymphocyte proliferation, thereby sustaining inflammation [122]. Pso-MSCs also exhibit a typical profile of cytokine expression (IFN-γ, IL-2, IL-17A/F, and IL-22), which unbalances the Th1/Th17 and Th2 profiles and upregulates the expression of VEGF/VEGF receptors and inducible nitric oxide synthase (iNOS) [123]. The intricate network of inflammation mediators is complicated by certain adipokines that worsen the Pso clinical course. Among these, leptin and resistin are the most extensively studied and are recognized as key signaling transducers linking obesity to psoriasis development. Although the precise mechanisms by which obesity contributes to Pso remain incompletely understood, elevated adipokine levels observed in patients promote the secretion of pro-inflammatory cytokines by KCs, thereby correlating with increased disease severity [84,120]. Among the various dysregulations of the IL-23/Th17 axis in Pso, there is an additional one impacting the high levels of leptin found in Pso patients. Leptin has been shown to increase the expression of IL-6, CXCL1, IL-8, monocyte chemoattractant protein-1 (MCP-1), and intercellular adhesion molecule-1 (ICAM-1) in dermal fibroblasts. Furthermore, leptin promotes the production of amphiregulin, a member of the epidermal growth factor (EGF) family, which drives the autocrine proliferation of KCs. Thus, high levels of leptin maintain inflammation in Pso, integrating obesity-related mechanisms with immune cell activity and the progression of autoimmunity [84]. Under normal conditions, the secretion of cytokines is triggered by the activation of CD4^+^ T cells through the interaction of antigen with the major histocompatibility complex (MHC) II, followed by their differentiation into various secretory T helper (Th) cell subsets. It was proposed that leptin induces cytokine secretion via specific receptors (leptin receptor LEPR) that activate CD4^+^ T cells, similar to the antigen—MHC II complex. This mechanism outlines a pathway through which white adipose tissue, particularly in cases of obesity, produces elevated levels of leptin that, in turn, activate key immune cell populations and contribute to the exacerbation of psoriatic skin lesions [84,124]. A diagram illustrating the interrelationship between the main adipokines and pro-inflammatory cytokines, as well as their downstream effects, is presented in Figure 2.

Although it is generally known that the hyperproliferation of KC is one of the hallmarks of Pso, emerging evidence suggests that metabolism is essential in the pathogenesis of the disease, particularly alterations in the lipid metabolism of KC. At the same time, alterations in lipid metabolism have been documented in the psoriatic skin lesions and in plasma/serum of Pso patients [1].

Dysregulation of lipid metabolism in Pso also involves alterations in lipid receptors, which may serve not only as indicators of inflammation but also as potential therapeutic targets. Hence, the reduced expression of lipoprotein-related receptors 5 and 6 (LRP5/6) in the skin and blood of patients with Pso increases after narrowband-ultraviolet (UV)B therapy, suggesting an anti-inflammatory role for LRP5/6 in the pathophysiology of Pso [125]. A dysregulated lipid metabolism in Pso directly impacts the differentiation and the functions of Th cells subpopulations, especially Th17 cells. Lipid accumulation sustains inflammatory processes and contributes to abnormal keratinization of the skin, favoring the initiation and relapse of Pso [126]. Elevated levels of free fatty acids (e.g., palmitic acid, oleic acid) modulate Th1 and Th17 cellular responses and their associated cytokine profiles. Pharmacological suppression of these fatty acids has been shown to reduce Th17 differentiation, thereby attenuating inflammation and alleviating disease manifestations [127,128]. Recent studies using animal models of psoriatic inflammation have identified a distinct population of regulatory T cells expressing peroxisome proliferator–activated receptor gamma (PPARγ) (CD4^+^Foxp3^+^) within the skin. This population appears to mitigate inflammation by suppressing IL-17A–producing γδ T cells, which are recognized as key mediators of psoriatic pathology [129]. Interesting results were obtained in another experimental model using obese mice, where elevated levels of long-chain free fatty acids, in conjunction with PPARγ^+^ skin Treg cells, correlated with mitochondrial dysfunction, cellular lipotoxicity, and oxidative stress. These results suggest that modulating the anti-inflammatory profile of PPARγ^+^ skin Treg cells may offer therapeutic benefits in obesity-associated inflammatory skin disorders [129]. Another recent study using an animal model of obesity and psoriasis demonstrated that a high-fat diet exacerbates disease severity by promoting the expansion of IL-17–producing γδ T cells. This research group confirmed the data obtained by Sivasami *et al*. regarding PPARγ^+^ T reg cells in skin, which generally function to restrict inflammation, become exhausted under a high-fat diet, thereby diminishing their immunosuppressive capacity [130].

**Figure 3:**
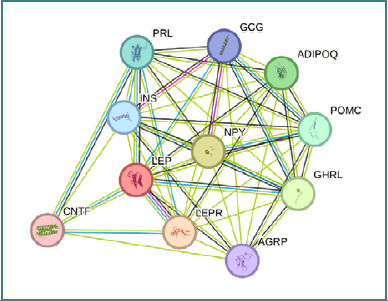
Leptin’s interactions as a key regulator of energy balance and body weight control. Once released into the circulation, leptin will bind to its receptor (LEPR), activating major signalling pathways (JAK2/STAT3 and MAPK cascades). It can bind to several important molecules such as neuropeptide (NP)Y, involved in the control of feeding and the secretion of gonadotropin-releasing hormone. It influences appetite-regulating hormones (ghrelin-obestatin preproprotein GHRL) and adiponectin (ADIPOQ), which are involved in the control of fat metabolism and insulin sensitivity. Additionally, it affects corticotropin-like intermediary peptide (POMC), which stimulates the adrenal glands to release cortisol, and prolactin (PRL), which acts primarily on the mammary gland by promoting lactation. Leptin (LEP) activates insulin A chain (INS) that decreases blood glucose concentration, increases cell permeability to monosaccharides, amino acids, and fatty acids, accelerates glycolysis, the pentose phosphate cycle, and glycogen synthesis in liver. It also influences glicentin-related polypeptide (GCG), a key mediator of glucose metabolism and homeostasis; agouti-related protein (AGRP), which regulates weight homeostasis; and ciliary neurotrophic factor (CNTF), a survival and regeneration factor for various neuronal cell types. (Network developed using STRING, version 12.0).

Adipokines modulate the functions of immune cells and their cytokine repertoire in Pso; among these, leptin, resistin, and adiponectin are key regulators of immune dynamics in autoimmunity [128]. Leptin, one of the most extensively studied adipokines, plays a key role in regulating energy balance and controlling body weight. Leptin is produced by subcutaneous adipose tissue, and inflammatory mediators such as TNF-α, IL-6, and IL-1β increase the production of leptin by adipocytes. Consequently, during inflammation leptin further activates granulocytes, monocytes/macrophages, DCs, and T lymphocytes, thus initiating Pso [124]. Additionally, the release of pro-inflammatory cytokines (IL-6, IL-1β, IL-12, TNF-α, and IL-17) is activated by increased leptin levels, favoring the onset of Pso once more. Moreover, high leptin levels in Pso modulate different signalling networks (e.g., Janus kinase [JAK]2/ signal transducer and activator of transcription [STAT]3 and mitogen-activated protein kinases [MAPK] cascade/FOS) impacting the immune landscape by endorsing CD4^+^CD25-T-cell proliferation and driving the differentiation of CD4^+^ memory T cells to a Th1 response. Leptin reduces autophagy associated with T-cell receptor engagement, a process considered essential for maintaining immune cell homeostasis [131,132]. A potential relationship between obesity and the onset of psoriasis is underscored by elevated leptin levels, which are linked to altered regulatory T cell (Treg) activity and disruption of the Treg/Th17 cell balance [125]. Figure 3 illustrates the interrelationships of leptin with key physiological processes and the molecules influenced by this adipokine.

Resistin is another important adipokine secreted by adipocytes, which has been identified in animal models and various human leukocytes [133]. It contributes to insulin resistance, diabetes mellitus, and obesity [134]. In Pso, like leptin, the plasma levels of resistin were found to be high, and recent studies propose resistin as a marker for treatment efficacy. Furthermore, a significant correlation has been observed between plasma resistin levels and Dermatology Life Quality Index (DLQI) scores in patients with Pso [135]. Preclinical reports suggest that resistin may induce Treg expansion, possibly through a mechanism involving reduced levels of IL-6, IL-12p40, and IL-23p19, a process governed by DCs [133]. Consequently, resistin has been proposed in several reports as having therapeutic potential in Pso due to its ability to modulate inflammatory cytokine production [136].

As underlined above, the antigen-presenting cell machinery in psoriatic lesions is represented by LCs that constitutively express high levels of CD1a and mediate lipid antigen presentation to CD1a-reactive T cells, inducing production of IL-22 and IL-17A. Furthermore, a significant source of neo-lipid antigens in the psoriatic inflammatory milieu is generated by phospholipase A2 (PLA2). The overexpression of PLA2 in Pso suggests potential therapeutic avenues, as both PLA2 inhibition and CD1a blockade may represent promising strategies for modulating disease activity [137].

In Pso, the central cellular players, KCs and Th lymphocytes, exhibit distinct metabolic features, including alterations in the tricarboxylic acid (TCA) cycle, amino acid metabolism, and fatty acid metabolism, combined with a dependence on glycolysis. Such metabolic particularities have served to define immunometabolism as an emerging tool for deciphering the etiopathogenesis of Pso. Consequently, long-term strategies for Pso management and improving patients’ quality of life could increasingly focus on modulating these metabolic pathways, potentially complemented by dietary interventions aimed at rebalancing metabolic disparities [138]. Several classes of lipids have been implicated in modulating the anti-inflammatory functions of MSCs as highlighted by recent studies. Using advanced lipidomic approaches, significant alterations were identified in the levels of phosphatidylcholine, phosphatidylethanolamine, phosphatidylserine, lysophosphatidylcholine, and sphingomyelin. In addition, a variation of these lipid groups occurs in the presence of TNF-α and IFN-γ, suggesting an active role of lipid metabolism in activating MSCs, contributing to their anti-inflammatory functions [139].

In patients with obesity, the behavior of MSCs is controlled by the secretion of adipokines. Alterations in adipokines, such as decreased levels of adiponectin and increased levels of leptin, nicotinamide phosphoribosyltransferase (NAMPT)/Visfatin, and/or resistin, trigger abnormalities in MSC functions that promote obesity-associated illnesses and alter the response to MSC therapy [140]. By targeting the adipokine network in obese patients or particularly obese-derived MSCs, their functionality and efficiency as therapeutic agents may be improved [140]. In several recent reports, it has been shown that the inflammatory milieu in Pso leads to a dysfunctional phenotype of MSCs governed by pro-inflammatory actions that contribute to the pathogenesis and maintenance of Pso [141,142]. Thus, MSCs induce KCs proliferation by producing high levels of pro-inflammatory factors that potentially activate phosphoinositide 3-kinase (PI3K)/protein kinase B (AKT) network, and concomitantly, MSCs inhibit KCs apoptosis by reducing caspase-3 levels [143,144]. Other studies indicate an alteration of KCs differentiation and a reduced epidermal turnover under dermal MSCs action. There is a self-sustaining vicious circle in MSCs, where the upregulation of the cellular myelocytomatosis oncogene (c-Myc), glucose transporter (GLUT)1, SCF, and EGF occurs after metabolic reprogramming is orchestrated by psoriatic KCs; this process results in increased proliferation of MSCs [145]. This cellular mechanism has an expected consequence, namely a disturbance in the Th1/Th17–Th2 balance. Additionally, the genes encoding the Th1 and Th17 cytokine repertoire become upregulated, thereby perpetuating the disease [146].

As in other skin pathologies, uncontrolled inflammatory responses triggered and maintained by increased adipose tissue [147,148] significantly contribute to the pathogenic profile of Pso. In this context, comprehensive efforts are required to mitigate the wide range of discomforts typically experienced by patients. Contemporary insights into the complex pathophysiology of psoriasis—where metabolic disorders emerge as key aggravating factors—must be leveraged to identify novel cellular and molecular networks. The ultimate goal is to suppress the persistent inflammatory state responsible for numerous complications, including treatment resistance during long-term therapy. A comprehensive vision for psoriasis research and clinical management should integrate established players in new roles, such as dermal mesenchymal stem cells, and revisit known mediators like lipid metabolism, to advance more effective therapeutic strategies.

## THERAPEUTIC INSIGHTS IN PSO – AN OPEN PERSPECTIVE

In accordance with its multifactorial pathogenesis, therapeutic approaches in Pso have been developed over many years of research in several lines of conduct. Some of the earlier approaches to Pso management included phototherapy and corticosteroid therapy. These two conventional treatments remain in clinical use, with phototherapy serving as a non-pharmacological option and corticosteroids continuing as a cornerstone for topical or systemic management. In 2022, roflumilast, a phosphodiesterase-4 (PDE4) inhibitor, was approved as a topical steroid-sparing agent [149]. Systemic agents, such as methotrexate (MTX) and cyclosporine (CyA), a calcineurin inhibitor, were introduced for the management of Pso in the late 1990s [150,151]. JAK inhibitors approved in 2021 modulate the immune signaling pathways and lack the renal and hepatic toxicity of CyA and MTX [152]. Interleukin 12/23 inhibitors [153], along with IL-17A blockers and IL-17 receptor antagonists [154], have recently revolutionized psoriasis treatment, and further investigations are ongoing to expand upon this therapeutic advancement. TNF-α inhibitors opened another therapeutic avenue for Pso, with numerous formulations extensively reviewed in 2025 [155]. In parallel, growing evidence highlights lipid metabolism as a reservoir for novel therapeutic insights in Pso management. Thus, an interesting therapeutic attempt reported by Caspary *et al*. involved inhibiting fatty acid oxidation by suppressing the activity of key enzymes related to this process. A significant enzyme is carnitine palmitoyltransferase (CPT)-1, which is highly active in psoriatic lesions compared to healthy skin. Inhibition of CPT-1 activity with specific agents, such as etomoxir, in animal models resulted in decreased epidermal thickness and reduced KC proliferation, highlighting CPT-1 as a promising metabolic target in psoriasis [156]. A lipidomic analysis of serum from patients with Pso revealed higher levels of saturated fatty acids and ω6 polyunsaturated fatty acids, alongside lower levels of monounsaturated fatty acids and ω3 PUFAs compared with healthy controls, indicating significant dysregulations in longchain fatty acid (LCFA) metabolism. Patients analyzed before and after mAb therapy showed a partial normalization of dysregulated LCFA profiles, underscoring the potential of metabolic profiling to provide valuable insights into disease mechanisms and therapeutic responses in psoriasis [157].

## CONCLUSION

Investigating fatty acid metabolism may open new opportunities for treating Pso, as immune cells utilize diverse metabolic pathways to sustain their functions. Thus, metabolic explorations could shape an adjuvant reprogramming approach to assist psoriatic patients undergoing biologic therapy. Networks of soluble factors, along with immune and non-immune cells, mediate the MSCs-inflammatory state in Pso. In this context, a vicious cycle between MSCs and KCs in psoriatic skin has been identified, resulting in a cascade of cellular and molecular events that fuel the progression of this autoimmune disease.
